# Supplementary material for: Sound advice: A calibration framework for defining detection space in passive acoustic monitoring
Source: PLoS One. 2026 Oct 27;21(10):e0350789. doi: 10.1371/journal.pone.0350789 (PMC13712539; doi:10.1371/journal.pone.0350789)
Supplement: Supplementary file 1 [file pone.0350789.s003.docx]

**Journal Requirements**

**1. Please ensure that your manuscript meets PLOS ONE style requirements, including those for file naming.**

***Response:*** *Thank you. We have reviewed the manuscript formatting and reference list and have prepared the revised files in accordance with the PLOS ONE submission requirements.*

**2. In your Methods section, please provide additional information regarding the permits obtained for the work, including the full name of the authority that approved field site access or a statement explaining why permits were not required.**

***Response:*** *Thank you. Field playback experiments were conducted in privately managed agricultural fields with verbal consent from the farmers. As the sites do not fall within the jurisdiction of the reserve forest, permits were legally not required for this study.*

We have now added a section on permits in the methods, and lines 250–252 now read “The study was conducted in privately owned agricultural fields and therefore, no permits were required beyond the verbal approval of the farm owner to enter their land.”

**3. Please remove funding-related text from the manuscript; funding information should appear only in the Funding Statement in the online submission form.**

***Response:*** *Thank you. We have ensured that funding information is not included as a separate funding statement in the manuscript and will provide the relevant funding information through the online submission form.*

**4–5. Please ensure that the grant information in the Funding Information and Financial Disclosure sections matches, and expand “DST” to the full name of the funder.**

***Response:*** *Thank you. We will ensure that the grant numbers are consistent across the Funding Information and Financial Disclosure sections and that DST is expanded to its full name in the cover letter and online submission information.*

**6. Please revise the Data Availability Statement so that the contact point for restricted/proprietary data is a non-author institutional contact and include the third-party organization contact information and full citation for the data.**

***Response:*** *Thank you. We will revise the Data Availability Statement in the submission system to provide an appropriate non-author institutional contact. We have also now supplied the data used in the MS in the supplementary table.*

**7–8. Please evaluate any reviewer-recommended citations for relevance and review the reference list for completeness, correctness, and any retracted publications.**

***Response:*** *Thank you. We have carefully reviewed and updated the citations and reference list, including correcting the citation numbering and adding references for the software and R packages used in the analyses.*

**Additional Editor Comments**

**Abstract. Western Ghats megadiversity is not necessarily widely known worldwide, and it would be helpful to add a sentence introducing this topic and then explain the need for users of PAM technology to adapt their study designs to the varied landscapes in the region.**

***Response:*** *Thank you. We interpret this comment as a reference to the introduction section as we do not mention the Western Ghats in the Abstract. We have now elaborated the introduction under “Monitoring megadiversity hotspots" section and added lines 93–96: "Over the last decade, the use of PAM has been rapidly expanding in megadiverse regions such as the Western Ghats in India. The mountain chain running parallel to India’s western coast is a global biodiversity hotspot characterized by high species richness and endemism, with landscapes spanning a wide range of elevations, vegetation types, and human-modified habitats." Then later added lines 108–112: "Therefore, determining the detection space in PAM studies in the megadiverse and heterogeneous landscapes such as the Western Ghats is critical because such environmental heterogeneity can alter sound propagation and detection, requiring the study designs to account for habitat-specific variation in the detection space of recorders."*

**Line 16: “synergistic” probably should be “interactive”.**

***Response:*** *Thank you. We have changed “synergistic” to “interactive”.*

**Line 313: “We provide the first systematic evidence…” Many effects of frequency, temperature, and humidity on signal detectability are well documented in the literature. Possibly, “the first” should be deleted.**

***Response:*** *Thank you. We agree that the original wording could be interpreted as overstating the novelty of these effects. We have removed “the first” and the revised sentence (line 344) now reads:*

*“We provide systematic evidence that detection probability in tropical agroecosystems is not simply a function of distance, but a frequency-dependent detection space shaped by ambient masking.”*

**Line 408: “even” possibly should be “uniform”.**

***Response:*** *Thank you. We have changed “even” to “uniform”.*

**The reviewer agrees that the organization of the Decision Support Framework can provide useful guidance in future studies.**

***Response:*** *Thank you. We appreciate this feedback.*

**Line 463: The sentence including “both signalers and the sensitivity” possibly needs to be rewritten. Are there two signalers? Sensitivity is not a noun.**

***Response:*** *Thank you for pointing this out. We have revised the sentence to clarify that we are referring to the acoustic spread of the signal source and the sensitivity of the recording sensor. The intended wording is (line 501): “Thus, accounting for the acoustic spread of signalers as well as the sensitivity of sensors will be critical for high-precision density estimation.”*

**Reviewer 1**

**General comment: The study is well written and valuable, but the limitation of using observer detection rather than SNR/in-band power or automated classification should be discussed more prominently, particularly given the increasing use of automated detection in PAM.**

***Response:*** *Thank you for this important point. We agree that observer-based detectability should not be interpreted as equivalent to automated classifier performance. We have clarified this in the “Acoustic Data Processing” section and substantially expanded the “Future Directions” section. We now explain that SNR and in-band power were not consistently well-defined under our field conditions because ambient noise varied substantially and signals at larger distances were often intermittent or fragmented. We therefore focused on observer-based operational detectability using five independent assessments and modeled the number of positive detections as a binomial response. We now explicitly state that these estimates are measures of observer-based detectability rather than direct proxies for automated classifier performance. We also discuss that automated detectors may respond differently to SNR, amplitude, frequency-specific energy, background noise, classifier architecture, training data, and decision thresholds, and recommend that future calibration studies should evaluate these recordings using both physical acoustic metrics and automated classifiers.*

*Line 212-215 now read “While signal-to-noise ratio (SNR) and in-band power are standard physical metrics used by automated classifiers for detectability in long-term monitoring data, they do not necessarily capture operational detectability under all recording and analysis conditions in a complex agroecosystem.”

Line 223-225 read: “Consequently, the detection probabilities estimated here should be interpreted primarily as measures of observer-based detectability rather than as direct proxies for automated classifier performance.”

Line 515-524 in the discussion section is “However, this measure should not be considered equivalent to detectability by automated classification algorithms. Automated detectors may respond differently to changes in SNR, signal amplitude, frequency-specific energy, and background noise, and their performance can also depend on classifier architecture, training data, and decision thresholds. Thus, while our observer-based measure captures an important component of effective detectability in PAM, it does not directly evaluate automated classifier performance. Future calibration studies could complement observer-based detection with SNR and other physical acoustic metrics and evaluate the same recordings using automated detection or classification algorithms to determine how these measures translate to machine-based detectability.”*

**Lines 92–95: If word limit becomes an issue, these study descriptions can be omitted and just citing the studies will suffice.**

***Response:*** *Thank you for the suggestion. As the manuscript remains within the permitted length and these examples provide useful context for the range of PAM applications in the Western Ghats, we have retained the brief study descriptions.*

**Line 94: The citation numbers in the text do not match the references throughout the manuscript. Please recheck all citations.**

***Response:*** *Thank you for spotting this. We have carefully rechecked and corrected the citation numbering throughout the manuscript and updated the reference list accordingly.*

**Lines 107–114: Briefly mention the broad goal of the study, the predictors tested (frequency, distance, recorder orientation, and environmental variables), and the a priori predictions.**

**Response:** *Thank you. We have revised the final paragraph of the Introduction to state the broad goal and the predictors tested. We now specify that we quantified how signal detectability changes with distance and how this relationship is modified by frequency, recorder orientation, ambient temperature, relative humidity, and wind speed. We also added our a priori expectations: detectability would decline with distance, higher frequencies would be more strongly affected; because the Song Meter Micro microphone is specified as omnidirectional, we expected little or no orientation effect; and we expected environmental conditions to influence signal detectability.*

*Line 122-133 now read “Our broad goal was to quantify how signal detectability changes with distance and to determine the extent to which this relationship is modified by signal frequency, recorder orientation, and local environmental conditions. Specifically, we determined the change in detection probability across frequencies ranging between 1–8 kHz over a 50–300 m distance relative to the ARU and examined the effects of ambient temperature, relative humidity, and wind speed. In addition, we used a combination of four ARUs to determine how the direction of the source affects the probability of signal detection. We expected a frequency-dependent decline in detectability over increasing distance, with higher frequencies being the most affected. As the Song Meter Micro microphone is specified as omnidirectional, we expected little or no effect of recorder orientation on detection probability. We also expected environmental conditions to influence signal detectability over increasing distance from signal source.”*

**Lines 119–120: How were these sites chosen randomly?**

***Response:*** *Thank you for pointing that out. The sites were not chosen “randomly” in a systematic way but were random relative to each other. We made sure that the sites were post-harvest paddy fields relative to one another while ensuring that each site provided a 500 m straight, unobstructed transect without major obstacles such as buildings, trees, or shrubs, which was necessary for the playback experiment. Line 147-148 now reads “We chose sites random relative to each other but ensured there was a 500 m straight line, without any major obstacles such as buildings, trees, or shrubs (Fig 1C).”*

**Line 125: Also mention the land area in SI units.**

***Response:*** *Thank you. We have revised the description of the paddy fields to report their area in hectares (0.25–0.41 ha).*

**Line 128: Again, how were the sites chosen randomly? If there was no systematic way, omit from the text.**

***Response:*** *Thank you. We have clarified the selection criteria and removed it from the text.*

**Line 142: Cite Audacity.**

***Response:*** *Thank you. We have now cited Audacity and added its reference to the reference list.*

**Line 145: Including a photo of the megaphone setup in the figure would be helpful, although optional.**

***Response:*** *Thank you for this great suggestion. Unfortunately, we do not have a high-quality photograph of the setup and therefore are unable to include this figure.*

**Line 148: Was the SPL meter using A-weighting (dBA)? If so, how did A-weighting influence playback standardization across frequencies? Does the meter have a relatively flat frequency response over the tested range? These potential sources of bias should be acknowledged and discussed.**

***Response:*** *Thank you. The SPL meter used in our experiments records using A-weighting, resulting in lower SPL corresponding to frequencies below 500 Hz and above 6 kHz. All frequencies tested in our experiments are within a frequency range that is minimally affected by A-weighting except for 8 kHz. While A-weighting could have been a source of bias when standardizing SPL for pure tones at 8kHz. We did not quantify this in our experiments. We have acknowledged the limitation in the revised manuscript. We have also updated the units of measured SPL values to dB(A).*

*Lines 165–172 now read “Each playback was standardized to a sound pressure level (SPL) between 78–82 dB (A) at 1 m from the speaker using a handheld sound level meter (Real Instruments HT 80A) at each distance and signal frequency. The SPL meter records using A-weighting, which mimics human perception of sound, resulting in a reduction of SPL corresponding to frequencies lower than 500 Hz and higher than 6 kHz. While this could potentially be a source of bias in standardizing playbacks, we relied on it as it was the only available recorder, and since our initial data transcription involved human participants aurally detecting signals from recordings.”*

**Line 159: Good idea.**

***Response:*** *Thank you. We appreciate the positive feedback.*

**Line 189: Insects are not always ambient noise. What if you want to monitor insects?**

***Response:*** *Thank you. We agree. We have revised the “Acoustic Data Processing” section so that insects are no longer presented categorically as ambient noise. The revised text refers more generally to transient ambient noise from wind and anthropogenic activities, while the Decision Support Framework also recognizes insects as potential high-frequency focal taxa.*

*Line 216 now reads “In these landscapes, transient ambient noise from wind and anthropogenic activities can fragment signals without necessarily rendering them undetectable to a trained observer or a classification algorithm”*

**Lines 214, 217: Cite these packages.**

***Response:*** *Thank you. We have now cited the R packages used in the analyses, including lme4, car, DHARMa, ggeffects, and emmeans, and added their corresponding references to the reference list.*

**Lines 226–229, 285–286, 315–316: Why have you not discussed the 8 kHz results? The decline in detectability from 4–6 kHz to 8 kHz is particularly interesting and is consistent with greater attenuation at higher frequencies.**

***Response:*** *Thank you for highlighting this omission. We have now incorporated the 8 kHz results throughout the Results and Discussion and expanded Table 2 to report the 8 kHz fixed effect and its interactions. The 8 kHz signal had significantly higher baseline detection odds than the overall mean, but its detectability was lower than the peak observed at 4–6 kHz. We now explicitly describe the decline at 8 kHz as evidence that the relationship between frequency and detection space is non-linear and discuss the reduction as being consistent with increasing attenuation toward the upper end of the tested frequency range. We also revised the discussion of the Song Meter Micro response to note its greater sensitivity at intermediate frequencies and reduced amplitude response toward both lower and upper ends of the tested range.*

*Line 258–261 “Specifically, the 6 kHz signal had the highest increase in odds of detection, indicating it was detected most readily, whereas the 8 kHz signal also showed significantly higher baseline detection odds than the overall mean, although to a lesser extent than 6 kHz (Table 2).”*

*Line 265–267: “Overall, marginal probabilities indicate that higher frequencies maintained a higher effective detection probability than lower frequencies as distance to source increased, although detection at 8 kHz declined relative to the peak observed at 4–6 kHz (Fig 2).”*

*Line 316–319 in the Discussion section now reads: “The 50% detection threshold for low-frequency signals (1 kHz) is approximately 100 m, whereas mid-range frequencies (~6 kHz) remain reliably detectable up to 250 m. Detectability subsequently declined at 8 kHz, indicating a non-linear relationship between frequency and detection space.”*

*Also, in “Signal transmission and the environment” section, lines 348–351: “However, detectability declined again at 8 kHz relative to the 4–6 kHz range, suggesting that the greater persistence of mid-frequency signals did not extend uniformly across the upper end of the tested frequency range.”

And lines 360–364 read “As a result, 1 kHz signals were strongly affected by low-frequency masking and ground-related propagation effects, whereas signals between 4 and 6 kHz occupied an acoustic window in which detectability was greatest. The subsequent reduction at 8 kHz is consistent with increasing attenuation toward the upper end of the tested frequency range.”*

**Lines 320–326: Nicely done.**

***Response:*** *Thank you. We appreciate the positive feedback.*
