# Supplementary material for: Sound advice: A calibration framework for defining detection space in passive acoustic monitoring
Source: PLoS One. 2026 Oct 27;21(10):e0350789. doi: 10.1371/journal.pone.0350789 (PMC13712539; doi:10.1371/journal.pone.0350789)
Supplement: Peer review history

# Peer review: Decision Letter 0

**DOI:** 10.1371/journal.pone.0350789.r001

**Authors:** Richard Mankin

28 Jul 2026

Dear Dr. Seshadri,

Please submit your revised manuscript by Aug 27 2026 11:59PM. If you will need more time than this to complete your revisions, please reply to this message or contact the journal office at plosone@plos.org. When you’re ready to submit your revision, log on to https://www.editorialmanager.com/pone/ and select the ‘Submissions Needing Revision’ folder to locate your manuscript file.

- A letter that responds to each point raised by the academic editor and reviewer(s). You should upload this letter as a separate file labeled 'Response to Reviewers'.
- A marked-up copy of your manuscript that highlights changes made to the original version. You should upload this as a separate file labeled 'Revised Manuscript with Track Changes'.
- An unmarked version of your revised paper without tracked changes. You should upload this as a separate file labeled 'Manuscript'.

If you would like to make changes to your financial disclosure, please include your updated statement in your cover letter. Guidelines for resubmitting your figure files are available below the reviewer comments at the end of this letter.

If applicable, we recommend that you deposit your laboratory protocols in protocols.io to enhance the reproducibility of your results. Protocols.io assigns your protocol its own identifier (DOI) so that it can be cited independently in the future. For instructions see: https://journals.plos.org/plosone/s/submission-guidelines#loc-laboratory-protocols. Additionally, PLOS ONE offers an option for publishing peer-reviewed Lab Protocol articles, which describe protocols hosted on protocols.io. Read more information on sharing protocols at https://plos.org/protocols?utm_medium=editorial-email&utm_source=authorletters&utm_campaign=protocols.

As the corresponding author, your ORCID iD is verified in the submission system and will appear in the published article. PLOS supports the use of ORCID, and we encourage all coauthors to register for an ORCID iD and use it as well. Please encourage your coauthors to verify their ORCID iD within the submission system before final acceptance, as unverified ORCID iDs will not appear in the published article. *Only*  the individual author can complete the verification step; PLOS staff *cannot*  verify ORCID iDs on behalf of authors.

We look forward to receiving your revised manuscript.

Kind regards,

Richard Mankin, Ph. D.

Section Editor

PLOS One

Journal Requirements:

When submitting your revision, we need you to address these additional requirements.

1.Please ensure that your manuscript meets PLOS ONE's style requirements, including those for file naming. The PLOS ONE style templates can be found at

https://journals.plos.org/plosone/s/file?id=wjVg/PLOSOne_formatting_sample_main_body.pdf and

https://journals.plos.org/plosone/s/file?id=ba62/PLOSOne_formatting_sample_title_authors_affiliations.pdf

2. In your Methods section, please provide additional information regarding the permits you obtained for the work. Please ensure you have included the full name of the authority that approved the field site access and, if no permits were required, a brief statement explaining why.

3. Please note that funding information should not appear in any section or other areas of your manuscript. We will only publish funding information present in the Funding Statement section of the online submission form. Please remove any funding-related text from the manuscript.

4. We note that the grant information you provided in the ‘Funding Information’ and ‘Financial Disclosure’ sections do not match.

When you resubmit, please ensure that you provide the correct grant numbers for the awards you received for your study in the ‘Funding Information’ section.

5. Please expand the acronym “DST” (as indicated in your financial disclosure) so that it states the name of your funders in full.

This information should be included in your cover letter; we will change the online submission form on your behalf.

6. In the online submission form you indicate that your data is not available for proprietary reasons and have provided a contact point for accessing this data. Please note that your current contact point is a co-author on this manuscript. According to our Data Policy, the contact point must not be an author on the manuscript and must be an institutional contact, ideally not an individual. Please revise your data statement to a non-author institutional point of contact, such as a data access or ethics committee, and send this to us via return email. Please also include contact information for the third party organization, and please include the full citation of where the data can be found.

7. If the reviewer comments include a recommendation to cite specific previously published works, please review and evaluate these publications to determine whether they are relevant and should be cited. There is no requirement to cite these works unless the editor has indicated otherwise.

8. Please review your reference list to ensure that it is complete and correct. If you have cited papers that have been retracted, please include the rationale for doing so in the manuscript text, or remove these references and replace them with relevant current references. Any changes to the reference list should be mentioned in the rebuttal letter that accompanies your revised manuscript. If you need to cite a retracted article, indicate the article’s retracted status in the References list and also include a citation and full reference for the retraction notice.

Additional Editor Comments:

Abstract. Western Ghats megadiversity is not necessarily widely known worldwide, and it would be helpful to add a sentence introducing this topic and then explain the need for users of PAM technology to adapt their study designs to the varied landscapes in the region.

Line 16 “synergistic” probably should be “interactive”

Line 313 “We provide the first systematic evidence that detection probability in tropical agroecosystems is not a linear function of distance, but a frequency-dependent detection space shaped by ambient masking.” Many effects of frequency, temperature, and humidity on signal detectability are well documented in the literature, some of which is referenced herein and the statement could be misinterpreted. Possibly, “the first” should be deleted.

Line 408 “even” possibly should be “uniform”

The reviewer agrees that the organization of the Decision Support Framework can provide useful guidance in future studies.

Line 463 The sentence including “both signalers and the sensitivity” possibly needs to be rewritten. Are there two signalers? Sensitivity is not a noun.

[Note: HTML markup is below. Please do not edit.]

Reviewers' comments:

Reviewer's Responses to Questions

**Comments to the Author**

1. Is the manuscript technically sound, and do the data support the conclusions?

Reviewer #1: Yes

**********

2. Has the statistical analysis been performed appropriately and rigorously?

Reviewer #1: Yes

**********

3. Have the authors made all data underlying the findings in their manuscript fully available?

The PLOS Data policy

Reviewer #1: Yes

**********

4. Is the manuscript presented in an intelligible fashion and written in standard English?

Reviewer #1: Yes

**********

Reviewer #1: This study examines how various environmental and experimental conditions such as temperature, humidity, recorder orientation, and distance from the sound source influence the detection space of ARUs in an Indian agroecosystem. It also proposes a decision-support framework to help PAM practitioners and researchers incorporate these environmental and hardware constraints into their study designs.

The authors have found several interesting patterns, including a nonlinear relationship between signal frequency and detectability, as well as a strong effect of recorder orientation. Overall, I found the manuscript to be well written, comprehensive, and easy to follow. Given the rapidly expanding use of passive acoustic monitoring (PAM) in ecological research and conservation, this study addresses an important methodological gap by highlighting the need to systematically quantify hardware and environmental constraints that influence detection. The manuscript is also prescriptive and provides a useful framework that researchers can implement when designing PAM studies.

I understand the authors' decision to use observer detection rates rather than signal-to-noise ratio (SNR) as a measure of operational detectability. However, I believe the study would have been even more relevant to contemporary PAM workflows if it had evaluated the performance of automated classification algorithms on the playback recordings. As automated detection is becoming increasingly central to long-term acoustic monitoring, one could argue that metrics such as SNR and in-band power are ultimately more relevant as they determine the detectability of signals by automated classifiers rather than by human annotators. Thus, I encourage the authors to discuss this limitation more prominently (eg. between lines 191-194 and in the discussion section), as it is important when considering the broader applicability of their findings.

That said, this limitation does not diminish the overall value of the study. I believe this manuscript represents an important contribution to the PAM literature and is suitable for publication following some revisions in the text. My suggestions/comments are mentioned below.

92-95: If word limit becomes an issue, these study descriptions can be omitted and just citing the studies will suffice.

94: The citation numbers in the text do not match the references throughout the manuscript. Eg. Arvind et al. (27) is (1) in the references section. I am not sure if this is intentional, but if not, please recheck all citations.

107-114: In this section, briefly mention 1) the broad goal of this study, 2) the predictors tested (e.g. frequency, distance, recorder orientation, and environmental variables) along with your a priori predictions. This would provide a clearer rationale for the analyses.

119-120: How were these sites chosen randomly?

125: Also mention the land area in SI units

128: Again, how were the sites chosen randomly? If there was no systematic way, omit from the text.

142: cite Audacity

145: this is optional, but including a photo of the megaphone setup in the figure would be helpful

148: A quick search of the SPL meter model suggests that measurements may have been recorded using A-weighting (dBA). Was this the case? If so, how did the A-weighting influence the playback standardization across the different frequencies? Additionally, does the SPL meter have a relatively flat frequency response over the tested range? Because SPL standardization is central to the study, I think these potential sources of bias should be acknowledged and discussed, even if they cannot be fully quantified.

159: Good idea

189: Insects are not always ambient noise. What if you want to monitor insects?

214, 217: cite these packages

226-229, 285-286, 315-316: Why haven't you discussed the 8 kHz results? I found this omission pervasive throughout the results and discussion (as well as Table 2). The decline in detectability from 4–6 kHz to 8 kHz is particularly interesting, as it is consistent with more attenuation expected at higher frequencies.

320-326: Nicely done.

**********

what does this mean?). If published, this will include your full peer review and any attached files.

If you choose “no”, your identity will remain anonymous but your review may still be made public.

**Do you want your identity to be public for this peer review?**  For information about this choice, including consent withdrawal, please see our Privacy Policy

Reviewer #1: **Yes:**  Vaibhav Chhaya

**********

[NOTE: If reviewer comments were submitted as an attachment file, they will be attached to this email and accessible via the submission site. Please log into your account, locate the manuscript record, and check for the action link "View Attachments". If this link does not appear, there are no attachment files.]

To ensure your figures meet our technical requirements, please review our figure guidelines: https://journals.plos.org/plosone/s/figures

You may also use PLOS’s free figure tool, NAAS, to help you prepare publication quality figures: https://journals.plos.org/plosone/s/figures#loc-tools-for-figure-preparation.

NAAS will assess whether your figures meet our technical requirements by comparing each figure against our figure specifications.

# Author comment: Author response to Decision Letter 1

**DOI:** 10.1371/journal.pone.0350789.r002

22 Aug 2026

We have provided a detailed response to reviewers document.

Attachment Submitted filename: Response to Reviewers.docx

# Editor report: Decision Letter 1

**DOI:** 10.1371/journal.pone.0350789.r003

**Authors:** Richard Mankin

24 Aug 2026

Sound Advice: A calibration framework for defining detection space in Passive Acoustic Monitoring

PONE-D-26-24644R1

Dear Dr. Seshadri,

We’re pleased to inform you that your manuscript has been judged scientifically suitable for publication and will be formally accepted for publication once it meets all outstanding technical requirements.

Within one week, you’ll receive an e-mail detailing the required amendments. When these have been addressed, you’ll receive a formal acceptance letter and your manuscript will be scheduled for publication.

An invoice will be generated when your article is formally accepted. Please note, if your institution has a publishing partnership with PLOS and your article meets the relevant criteria, all or part of your publication costs will be covered. Please make sure your user information is up-to-date by logging into Editorial Manager at Editorial Manager® and clicking the ‘Update My Information' link at the top of the page. For questions related to billing, please contact billing support.

If your institution or institutions have a press office, please notify them about your upcoming paper to help maximize its impact. If they’ll be preparing press materials, please inform our press team as soon as possible -- no later than 48 hours after receiving the formal acceptance. Your manuscript will remain under strict press embargo until 2 pm Eastern Time on the date of publication. For more information, please contact onepress@plos.org.

Kind regards,

Richard Mankin, Ph. D.

Section Editor

PLOS One

Additional Editor Comments (optional):

Reviewers' comments:

# Editor report: Acceptance letter

**DOI:** 10.1371/journal.pone.0350789.r004

**Authors:** Richard Mankin

PONE-D-26-24644R1

PLOS One

Dear Dr. Seshadri,

I'm pleased to inform you that your manuscript has been deemed suitable for publication in PLOS One. Congratulations! Your manuscript is now being handed over to our production team.

At this stage, our production department will prepare your paper for publication. This includes ensuring the following:

* All references, tables, and figures are properly cited

* All relevant supporting information is included in the manuscript submission,

* There are no issues that prevent the paper from being properly typeset

You will receive further instructions from the production team, including instructions on how to review your proof when it is ready. Please keep in mind that we are working through a large volume of accepted articles, so please give us a few days to review your paper and let you know the next and final steps.

Lastly, if your institution or institutions have a press office, please let them know about your upcoming paper now to help maximize its impact. If they'll be preparing press materials, please inform our press team within the next 48 hours. Your manuscript will remain under strict press embargo until 2 pm Eastern Time on the date of publication. For more information, please contact onepress@plos.org.

You will receive an invoice from PLOS for your publication fee after your manuscript has reached the completed accept phase. If you receive an email requesting payment before acceptance or for any other service, this may be a phishing scheme. Learn how to identify phishing emails and protect your accounts at https://explore.plos.org/phishing.

If we can help with anything else, please email us at customercare@plos.org.

Thank you for submitting your work to PLOS One and supporting open access.

Kind regards,

PLOS One Editorial Office Staff

on behalf of

Dr. Richard Mankin

Section Editor

PLOS One
